# Drug Checking as Strategy for Harm Reduction in Recreational Contests: Evaluation of Two Different Drug Analysis Methodologies

**DOI:** 10.3389/fpsyt.2021.596895

**Published:** 2021-02-22

**Authors:** Martina Fregonese, Andrea Albino, Claudia Covino, Alessio Gili, Mauro Bacci, Alessia Nicoletti, Cristiana Gambelunghe

**Affiliations:** ^1^Harm Reduction Services, Cooperative “Borgorete”, Perugia, Italy; ^2^Local Health Unit, USL Umbria 1, Ser.T Perugia, Ospedale S. Maria della Misericordia, Perugia, Italy; ^3^Hygiene and Public Health Section, Department of Medicine and Surgery, University of Perugia, Perugia, Italy; ^4^Forensic Medicine, Forensic Science and Sports Medicine Section, Department of Medicine and Surgery, University of Perugia, Perugia, Italy

**Keywords:** drug checking, harm reduction, club drugs, colorimetric test, gas chromatography/mass spectrometry

## Abstract

**Introduction:** Drug checking as a part of drug harm-reduction strategies represents an essential aspect of public health policies. It focuses on rapid identification of drugs that individuals intend to use during night events, in order to implement health-protective behaviors. Chemical drug analysis techniques vary considerably, from simple colorimetric reagents to advanced forensic methods such as gas chromatography/mass spectrometry (GC/MS).

**Materials and Methods:** In 2019, drug-check services were offered at some night events in Umbria (Central Italy). One hundred and twenty attendees directly delivered unidentified substances to a harm-reduction worker, who collected a few milligrams of the substances on ceramic plates and added a drop of colorimetric reagent. Multiple reagents were used to increase the diagnostic capacity of a substance, which may react with a specific drug or a few drugs. Later, a fraction of the samples was analyzed by GC/MS. The concordance of the results obtained using these two methodologies and the intended behaviors of consumers after being informed of the test result was evaluated.

**Results:** We analyzed 120 samples by colorimetric test: 32 MDMA, 25 ketamine, 10 amphetamine, 11 cocaine, 8 heroin, and 4 LSD samples. The results were inconclusive for 29 samples. The GS/MS analysis confirmed MDMA in 84%, ketamine in 78%, amphetamine in 91%, cocaine in 92%, heroin in 88%, and LSD in 100% of the samples. The results of samples with inconclusive results were as follows: 2, MDMA; 7, ketamine; 2, amphetamine; 2, cocaine; 2, heroin; 2, mephedrone; 6, mixes; 1, debris; and 5, adulterants as the main component. Twenty-one of 29 participants reported that they had no intention of consuming the unidentified substance.

Discussion: The high percentage of individuals who claimed no intention of consuming the unidentified drugs indicates that drug checking is viable as a part of drug harm-reduction strategies. Overall, colorimetric reagents showed a good performance with regard to samples being unadulterated (LSD) or minimal in quantity, but failed to identify mixtures of substances and the adulterants present in them. Therefore, the use of more discriminatory on-site methods such as Raman or infrared spectrometry is strongly recommended.

## Introduction

Illicit drug use is common among attendees of clubs and night events (such as bars, discos, parties, and music festivals); these individuals are at a higher risk of using drugs than the general population (1–4). The most commonly used “club drugs” (also known as “party drugs” or “recreational drugs”) include entactogens such as methylenedioxymethamphetamine (MDMA, Ecstasy); sedatives such as flunitrazepam (Rohypnol) and gamma-hydroxybutyrate (GHB); stimulants such as amphetamine, cocaine, and methamphetamine; and hallucinogens such as ketamine and lysergic acid diethylamide (LSD) (1, 5, 6). These drugs have nicknames that change over time. The use of these drugs tend to be highly prevalent among night event attendees (3–7). Club drugs are used to increase the performance and enjoyment of recreational events, reduce physical fatigue, increase the communication and relational skills of individuals, and increase or modify the perception of reality (8). These drugs act on the central nervous system involving different neuromodulating systems and have different pharmacologic properties, physiological and psychological effects, and potential consequences (6). MDMA and stimulant drugs act mainly on dopaminergic, noradrenergic, and serotonergic neurons; flunitrazepam enhances the action of the neurotransmitter GABA; and hallucinogens such as ketamine and LSD act as an NMDA receptor antagonist and activator of serotonin 2A (5-HT2A) receptor, respectively (6, 9). Several studies have highlighted that the use of club drugs is associated with serious physical health problems (e.g., hyperthermia, convulsions, and multiorgan failure) (3), risky sexual behavior (10), and violence and crime (4). Stimulant intoxication can manifest as mania or hypomania, and substance withdrawal often manifests as dysphoria and depression (11). Cocaine and amphetamines may mimic bipolar spectrum disorders, causing symptoms such as euphoria, increased energy, and decreased appetite (11, 12). Psychotic symptoms can also be observed (11, 12). Individuals who regularly use GHB often present with mild-to-moderate symptoms, including anxiety, irritability, mood swings, aggression, insomnia, and hallucinations (13). Repeated and prolonged use of ketamine can lead to chronic memory impairment, hallucinations, confusion, and “word blocking” (13). LSD-associated psychiatric disorders can include psychosis and persistent hallucinations (13). Club drugs are consumed worldwide, especially in the nightlife scene. In 2018, MDMA consumption in the European Union was estimated to be approximately 2.6 million in the adult population and 2.1 million among young adults; the corresponding numbers for amphetamines were 1.7 and 1.2 million, respectively (14). The consumption of MDMA for the same year in the USA was estimated to be 7.30, 0.80, 10.50, and 7.50% among individuals aged 12 years or higher, 12–17 years, 18–25 years, and 26 years or higher, respectively (15). The corresponding percentages for methamphetamine were estimated to be 1, 0.30, 2.50, and 6.50%, respectively (15).

Several international projects [Nightlife Empowerment & Well-being Implementation Project; Drug Checking Service: Good Practice Standards; Trans European Drugs Information (TEDI) Workgroup; Factsheet on Drug Checking in Europe, 2011; European Monitoring Center for Drugs and Drug Addiction; and An Inventory of On-site Pill-Testing Interventions in the EU: Fact Files, 2001] have implemented several harm-reduction strategies to prevent recreational drug use among young people (16). The strategies encompass interventions, programs, and policies that seek to reduce the health-related, social, and economic harms of drug use to individuals, communities, and societies (1). They are aimed at ensuring a pragmatic manner of dealing with drug use through a hierarchy of intervention goals that emphasizes on reducing the health-related harms of continued drug use, offering, for example, opioid substitution treatment and needle and syringe programs to prevent death due to overdose and reduce the spread of infectious diseases (1, 17). Since the 1960s, harm-reduction services are occasionally available at various types of nightly musical events to inform users about the risks of drug use and ways of risk minimization (18). Such services are called “street drug analysis,” “pill testing,” “drug checking,” “adulterant screening,” “drug testing,” and “multi-agency safety testing (19).” Their main purpose is to provide individual drug users free testing services to identify the drugs that they intend to use during an event and all possible information on substance purity. This is to ensure that the users have the option to make a more informed choice about substance use (2, 20). The main objectives of this type of harm-reduction strategies are to change consumer behavior at the time of consumption, that is, when a consumer is confronted with an unexpected test result, facilitate brief interventions and referrals to services, and/or inform clinical management (20).

Two broad categories of drug-check services are offered. The most common are color reagents, Fourier transform infrared spectroscopy, ultraviolet-visible spectroscopy, and Raman spectroscopy (21). The most widely used on-site drug-checking method is the use of simple color reagent test kits (Marquis reagent and others). These tests are purely presumptive in nature although they can be fairly accurate in identifying a compound and/or mixture when a standardized procedure comprising a series of tests is used (16, 21). Furthermore, color reagent tests are rapid and relatively inexpensive, and in most instances, high-level scientific knowledge is not required to perform these tests and interpret the findings (16). The current gold standards in forensic drug analysis are chromatographic techniques such as high-performance liquid chromatography or gas chromatography (GC) coupled with mass spectrometry (MS), wherein a sample is compared with a reference library of known substances including a wide range of adulterants (16, 22, 23). These techniques are highly discriminative and quantitative but are not rapid, unlike colorimetric tests; furthermore, they are associated with a high cost. Additionally, highly qualified personnel are required for their execution (16).

Despite a consolidated body of literature that shows the effectiveness of drug-checking initiatives for public health surveillance (24–27), there are criticisms about the limitations of these initiatives and the potential false sense of security that might be related to the use of color reagent test kits. To address the latter issue, the use of more discriminatory testing methods that may provide more reliable results is recommended (20, 24, 28).

The aim of this study was to compare the results of drug checking performed using colorimetric tests during five night events in Umbria, a region in Central Italy, in 2019, with those of an established forensic gas chromatography/mass spectrometry (GC/MS) method, in order to assess the concordance between the results obtained using these methodologies. The drug-checking service based on forensic methodology allowed the determination of the main component of samples, as well as adulterants and contaminants, which are of particular concern because they might result in adverse health consequences (29). Furthermore, we could evaluate the presence of new psychoactive substances (NPS) that may be circulating in recreational contests in central Italy.

## Materials and Methods

### Setting

Drug-checking services were offered during five night events in Umbria in 2019. Individuals at these events were informed about the availability of on-site, anonymous no-cost drug-checking service via word-of-mouth or service promotion. In total, 120 attendees of the events, that is, approximately 15% of the total number of attendees, participated in the study by visiting the service camp to obtain information on drugs and eventually requested an analysis of the substance that they intended to consume. They were then informed that the samples would be subjected to a colorimetric test on-site and a further laboratory test later. These procedures were performed after obtaining informed consent of the participants. The participants anonymously and spontaneously provided brief information about their age, expectation about the chemical nature of the sample, and their behavior in terms of drug use (i.e., whether the substance had been already consumed/not consumed before the test and whether they would consume it/not consume it after receiving the test result), using a completely anonymous and voluntary form (Supplementary Data Sheet 1). A few milligrams of the drug was analyzed on-site using colorimetric reagents. Furthermore, an anonymous fraction without any personal identifiable information of the collected sample was delivered to the Forensic Toxicology Laboratory of the University of Perugia (Supplementary Data Sheet 2) for a confirmatory analysis by GC/MS. Seventy-one of the 120 samples delivered were powders, 16/120 were tablets, 29/120 were crystals, and 4/120 were blotters. The operators conducting the test and interview had a specific training in harm reduction and maintaining the confidentiality of individuals. They worked under the supervision of the local health unit. A physician was present on-site. Both physician and operator were available to discuss the colorimetric test results and any potential health risks associated with substance use. In particular, the participants were informed of the limitations of colorimetric tests, including the inability to quantify purity, possible failure in detecting adulterants, and that the result did not guarantee the safety of the pill. As polyconsumption of substances is a rather common event, information was provided about the risks of combining alcohol with other drugs.

The data collected lacked any identifiable information, and data associated with the risk of re-identification of participants were excluded. The ethics committee of the University of Perugia Institutional Review Board approved this study (protocol no. 51855). According to General Data Protection Regulation (GDPR), the storage security of the anonymously collected data (demographic and analytical) was ensured such that it prevented any unauthorized access (via authentication and access control and use of passwords to access electronic files).

### Chemicals and Reagents

Methanol, hydrochloric acid, and ammonia solution were of analytical quality and purchased from Merck (Darmstadt, Germany). Certified reference standards of the target analytes (as free bases or salts) were supplied by Sigma Aldrich (St. Louis, MO, USA). The Hoffmann, Lieberman, Mandelin, Marquis, Merke, and Scott colorimetric reagents were produced by Chemical Safety sp.zo.o. (Warsaw, Poland).

### On-site Tests

The on-site procedure was performed according to an internal protocol of harm-reduction service developed in collaboration with the local health unit. The participants directly delivered the drug sample for testing on-site to a harm-reduction worker. Preliminary screening was performed by scraping off a small amount of the substance (4–8 mg). The substance obtained was divided into six aliquots of ~0.5–1 mg and placed on different ceramic plates. Thereafter, a drop of Hofmann, Lieberman, Mandelin, Marquis, Merke, and Scott colorimetric reagents, respectively, was added. Each reagent produces a color change after a chemical reaction between an illicit substance and the reagent. This result was then matched to a color chart showing the expected color change of the reagent with various illicit substances (30). Samples were reported to be “positive” if the color changes occurred in the range displayed on the reagent chart after the addition of Hofmann, Lieberman, Marquis, Mecke, Mandelin, and Scott reagents. If the reagent tests did not show results consistent with any listed substance, the substance was classified as “Unknown” (31). After being informed of the test results and limitations, the participants were asked if they still intend to consume the product with the option of answering as “Yes” or “No.” The results regarding possible unusual or concerning substances were posted outside the drug-checking camper and were shared with other national harm-reduction groups and regional health authorities.

### Forensic Analysis

**Figure 1:**
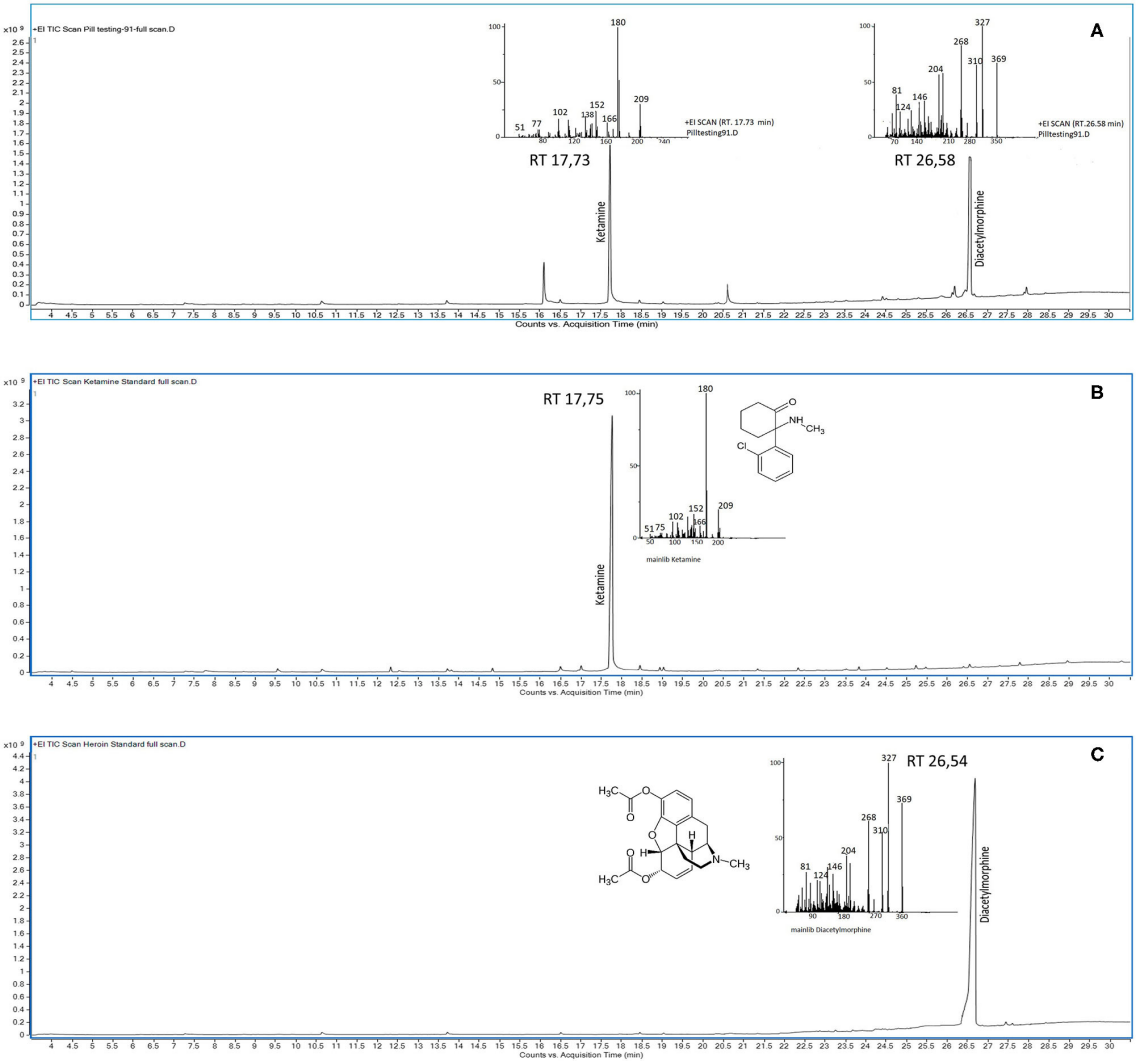
GC/MS chromatograms of **(A)** a drug sample containing a mixture of ketamine and heroin (diacetylmorphine) with the respective mass spectrum of the peaks at the retention time (RT) of 17.73 and 26.58 min, respectively; and **(B)** ketamine analytical standard with the relative mass spectrum of the peak at the RT of 17.75 min; and **(C)** heroin (diacetylmorphine) analytical standard with the relative mass spectrum of the peak at the RT of 26.54 min.

Each substance (~1 mg) anonymously submitted to the Forensic Toxicology Laboratory was extracted in three ways: (1) in methanol; (2) in methanol alkalized with ammonium solution; and (3) in methanol acidified with dilute hydrochloric acid. The extracts were sonicated for 10 min, and then injected into a triple quadrupole 7000C GC/MS system (Agilent Technologies) operated under the electron impact ionization (EI) mode and fitted with a 7890 B gas chromatograph (Agilent Technologies, Palo Alto, CA, USA). This system was equipped with an HP-5MS (Agilent Technologies, Palo Alto, CA, USA) capillary column (length 30 m, inner diameter 0.50 mm, and film thickness 0.25 mm), operated with helium at a flow rate of 1 ml/min and temperature programming of 80°C for 1 min ramped at 8°C/min to 300°C and held for 2 min. The samples (1 μl) were injected into a split–spitless injector at 250°C in the spitless mode (1 min). The ion source and AUX temperatures were 250 and 280°C, respectively. MS acquisition for unknown substances was performed in the full-scan mode in the range of 41–500 amu. Unknown substances were identified by matching experimental full-scan spectra against the NIST spectral library, the most powerful database for screening unknown substances. The retention times and relative mass spectra of the analytes detected were then compared with those of certified standards analyzed under the same experimental conditions (23). An example of a representative chromatogram of a sample containing a mixture of ketamine and heroin is shown in Figure 1.

### Data Analysis

Descriptive statistics including frequency, percentage, frequency table for categorical variables, and mean ± standard deviation (SD) for quantitative variables were calculated. Categorical variables were evaluated using χ^2^ or Fisher's exact test. Significance was set to the ≤0.05 level in all tests. Statistical analyses were performed using STATA v. 14.2 (StataCorp LP, College Station, TX, USA).

## Results

### Samples

Of the 120 attendees, 75.0% were men. The mean age of the attendees was 29.16 ± 5.99 (range: 16–44) years. Each participant requested us to analyze a substance of concern. Approximately half of the participants (43.33%) had not consumed the substance in their possession at the time of requisition.

### Colorimetric Test Outcomes and Participants' Behavioral Intentions

Of the 120 participants who requested drug checking, 85% reported that they expected to identify the substance purchased, 10% did not expect to identify the active substances but only adulterants, and 3.33% expected to identify substances different from those in his/her possession. Although there were no limits on the number of substances to be analyzed, each participant requested a test for only one substance. Therefore, 120 drug samples were analyzed using colorimetric tests. The results were obtained were conclusive and inconclusive for 91 (75.83%) and 29 (24.17%) samples, respectively; the former were classified as “positive” and the latter as “unknown.”

Table 1 shows the comparison between participant expectations about the substance purchased and the results obtained using colorimetric tests. Most of the participants requested to test whether a substance that they possessed was MDMA (*n* = 40), followed by ketamine (*n* = 34), cocaine (*n* = 14), heroin (*n* = 10), LSD (*n* = 4), and mescaline (*n* = 1). Based on the colorimetric tests, 33, 25, 10, 11, 8, and 4 samples were classified as positive for MDMA, ketamine, amphetamine, cocaine, heroin, and LSD, respectively. The colorimetric tests confirmed the presence of substance expected by the participants in 75% of the cases.

**Table 1:** Comparison between colorimetric test results and client expectations about the substance bought and submitted for testing.

**Client expectation (No. of samples)**	**Colorimetric test results (No. of samples)**							
	**Ketamine (*n* = 25)**	**MDMA (*n* = 33)**	**Amphetamine (*n* = 10)**	**Cocaine (*n* = 11)**	**Heroin (*n* = 8)**	**LSD (*n* = 4)**	**Mescaline (*n* = 0)**	**Unknown (*n* = 29)**
Ketamine (*n* = 34)	25	0	0	0	0	0	0	9
MDMA (*n* = 40)	0	32	0	0	0	0	0	8
Amphetamine (*n* = 17)	0	0	10	0	0	0	0	7
Cocaine (*n* = 14)	0	0	0	11	0	0	0	3
Heroin (*n* = 10)	0	0	0	0	8	0	0	2
LSD (*n* = 4)	0	0	0	0	0	4	0	0
Mescaline (*n* = 1)	0	1	0	0	0	0	0	0

Regarding the intended behavior of participants upon receiving the test result, the statistical analysis revealed no significant association (Pr = 0.160) among the substance that they thought they had bought, the outcome of the test, and the subsequent reuse of the substance. Thus, in most cases, the intention of individuals to use the substance was independent of the colorimetric test result (Table 2). In terms of details, 99/120 (82.50%) reported that they would consume the substance analyzed, where 21/120 (17.50%) reported as they would not consume it. In particular, 21/29 (72.41%) participants who received an “unknown” result from the colorimetric test declared their intention of not consuming the substance. Upon a detailed examination of the participants' intended behavior regarding the use of substances before and after the test, 9/120 participants declared that they had not used the substance before the test and did not want to use it after the test, 5/120 admitted to having used the substance before but not wanting to do it again, and 40/120 reported that they had not consumed the substance before the test but intended to do it later.

**Table 2:** Answers to the questions posed to users about the consumption of the substance before submission of the sample for the colorimetric test and users' intention to use the tested substance after receiving the colorimetric test result.

**Intention to use the substance after the test**	**Substance used before the test**		
	**No**	**Yes**	**Total**
NO	12	9	21
	57.14%	42.86%	100%
	23.08%	13.24%	17.50%
YES	40	59	99
	40.40%	56.67%	100%
	76.92%	92.42%	82.50%
Total	52	68	120
	43.33%	56.67%	100%
	100%	100%	100%

*Pearson chi2 (1) = 1.9768; Pr = 0.160*.

### Comparison of the Colorimetric Test Outcomes and GC/MS Results

A comparison of the colorimetric test outcomes and GC/MS results is shown in Table 3. The GC/MS analysis of the 120 drug samples revealed the presence of ketamine (32 samples), MDMA (28 samples), amphetamine (11 samples), cocaine (12 samples), heroin (seven samples), LSD (four samples), mephedrone (two samples), and methamphetamine (one sample). Furthermore, it was possible to identify nine samples with mixtures of substances, in which the substances were present in approximately equal quantities and had similar pharmacological activities. Thirteen samples contained multiple adulterants in approximately equal quantities. One sample was found to be free of active substances and was classified as “debris” (Table 3).

**Table 3:** Comparison between colorimetric test and GC/MS results.

**Colorimetric results (No. of samples)**	**GC/MS results (No. of samples)**										
	**Ketamine (*n* = 25)**	**MDMA (*n* = 28)**	**Amphetamine (*n* = 11)**	**Cocaine (*n* = 12)**	**Heroin (*n* = 7)**	**LSD (*n* = 4)**	**Mephedrone (*n* = 2)**	**MIX (*n* = 9)**	**Debris (*n* = 9)**	**Methamphetamine (*n* = 1)**	**Adulterant as main component (*n* = 13)**
Ketamine (*n* = 25)	25	0	0	0	0	0	0	0	0	0	0
MDMA (*n* = 33)	0	26	0	0	0	0	0	2	0	1	4
Amphetamine (*n* = 10)	0	0	9	0	0	0	0	1	0	0	0
Cocaine (*n* = 11)	0	0	0	10	0	0	0	0	0	0	1
Heroin (*n* = 8)	0	0	0	0	5	0	0	0	0	0	3
LSD (*n* = 4)	0	0	0	0	0	4	0	0	0	0	0
Unknown (*n* = 29)	7	2	2	2	2	0	2	6	1	0	5

Regarding the 29 samples that were classified as “unknown” based on the results of the colorimetric tests, the GC/MS analysis showed the following to be the main component in these samples: ketamine (*n* = 7), MDMA (*n* = 2), amphetamines (*n* = 2), cocaine (*n* = 2), heroine (*n* = 2), mephedrone (*n* = 2), mixtures (*n* = 6), debris (*n* = 1), and adulterants (*n* = 5). The nine drug mixtures identified using the GC/MS analysis as mixtures were present in approximately equal parts: ketamine with methamphetamine (*n* = 2) and heroin (*n* = 1), MDMA with methamphetamine (*n* = 1) and amphetamine (*n* = 2), amphetamine with methamphetamine (*n* = 1) and MDMA (*n* = 1), and cocaine with heroin (*n* = 1).

The analytical performance of the single reagents (Hofmann, Lieberman, Mandelin, Marquis, Merke, and Scott reagents) used on-site was also assessed (Table 4). LSD was correctly identified using its specific Hoffmann's reagent; 78% of the ketamine samples were identified using Liebermann and Mandelin reagents. MDMA was identified in 33 samples using its specific reagents (Liberman, Mandelin, Mecke, and Maquis reagents). However, of these, only 28 were confirmed by GC/MS. Cocaine was correctly determined in 92% of the samples using Scott and Liberman reagents. Heroin was identified in eight samples using Liberman, Marquis, Mecke, and Mandelin reagents, but was confirmed in only seven samples by GC/MS. The two mephedrone samples could not be identified with the specific reagent, Lieberman reagent.

**Table 4:** Colorimetric reaction of each substance with each reagent and number of samples in which the substance was identified by the colorimetric test and GC/MS.

**Substance**	**Colorimetric reagent**					
	**Lieberman**	**Mandelin**	**Marquis**	**Mecke**	**Scott**	**Hoffmann**
**Ketamine**						
Color reaction	Light yellow	Orange-Red	No reaction	No reaction	No reaction	No reaction
No. samples positive at CT	25	25				
No. samples confirmed at GC/MS	32	32				
	Proportion 0.78 S.E 0.073 95% C.I 0.60 ± 0.91	Proportion 0.78S.E 0.07395% C.I 0.60 ± 0.91				
**MDMA**						
Color reaction	Brown-Black	Blue-Black	Violet-Black	Green-Black	No reaction	No reaction
No. samples positive at CT	33	33	33	33		
No. samples confirmed at GC/MS	28	28	28	28		
	Proportion 0.84 S.E 0.062 95% C.I 0.68 ± 0.94	Proportion 0.84 S.E 0.06295% C.I 0.68 ± 0.94	Proportion 0.84 S.E 0.062 95% C.I 0.68 ± 0.94	Proportion 0.84S.E 0.06295% C.I 0.68 ± 0.94		
**Amphetamine**						
Color reaction	Orange	Green	Orange	No reaction	No reaction	No reaction
No. samples positive at CT	10	10	10			
No. samples confirmed at GC/MS	11	11	11			
	Proportion 0.91 S.E 0.087 95% C.I 0.59 ± 1.0	Proportion 0.91S.E 0.08795% C.I 0.59 ± 1.0	Proportion 0.91 S.E 0.087 95% C.I 0.59 ± 1.0			
**Cocaine**						
Color reaction	Light orange	No reaction	No reaction	No reaction	Blue	No reaction
No. samples positive at CT	11				11	
No. samples confirmed at GC/MS	12				12	
	Proportion 0.92 S.E 0.080 95% C.I 0.62 ± 1.0				Proportion 0.92 S.E 0.080 95% C.I 0.62 ± 1.	
**Heroin**						
Color reaction	Black	Green-Brown	Violet	Green	No reaction	No reaction
No. samples positive at CT	8	8	8	8		
No. samples confirmed at GC/MS	7	7	7	7		
	Proportion 0.88 S.E 0.12 95% C.I 0.47 ± 1.0	Proportion 0.88S.E 0.1295% C.I 0.47 ± 1.0	Proportion 0.88 S.E 0.12 95% C.I 0.47 ± 1.0	Proportion 0.88S.E 0.1295% C.I 0.47 ± 1.0		
**LSD**						
Color reaction	No reaction	No reaction	No reaction	No reaction	No reaction	Violet
No. samples positive at CT						4
No. samples confirmed at GC/MS						4
**Mephedrone**						
Color reaction	Yellow	No reaction	No reaction	No reaction	No reaction	No reaction
No. samples positive at CT	0					
No. samples confirmed at GC/MS	2					

*S.E., standard error; C.I., confidence interval; CT, colorimetric test*.

### Adulterants Detected

Although adulterants could not be identified through the on-site colorimetric tests, GC/MS could detect the presence of adulterants in 63/120 samples (52.50%), of which 53 (84.13%) contained commonly found adulterants and 10 (15.87%) contained unusual substances. The following adulterants were found in the cocaine samples: caffeine, levamisole, lidocaine, benzocaine, phenacetin, and acetaminophen. Paracetamol, diazepam, acetaminophen, and codeine were the adulterants in the heroin samples (32–34). MDMA and amphetamine samples contained caffeine, methamphetamine, MDA, and ephedrine as adulterants (31). LSD samples were found to be unadulterated. A few ketamine samples were found to be adulterated; the finding was in accordance with that of a previous study (29). The GC/MS analysis resealed adulterants not commonly found or not expected: piracetam, tramadol, and mephedrone in MDMA; methamphetamine, amphetamine and heroin in ketamine; and cocaine in heroin.

## Discussion

### Aim of Drug Checking

Several countries worldwide have implemented drug checking with the aim to provide targeted preventive messages to recreational drug users. This approach, which is more individualized than mass media campaigns, provides an incentive for drug users to participate in a dialogue about harm prevention and reduction, because they get to know the test results and receive information about the drugs they are consuming. Although there are arguments in favor of drug checking, the strategy has also met with criticisms concerning the technical limitations of color reagent test kits and whether such interventions are better than no intervention at all because of the false sense of security that pill testing would engender. Furthermore, the evidence to date is equivocal (20, 22, 35). Hence, in this study, we aimed to evaluate the analytical performance of colorimetric tests.

During the study, drug-checking services involving the use of on-site colorimetric tests were offered at five night events in Umbria in 2019. The drug checking point was indicated by signs placed at the entrance and at various points in the place of the event, with attention toward highlighting that the service was free and would be provided anonymously. This information was also spread by word-of-mouth among users. There was a collaboration between drug test operators and the police. The police were always informed of the service at the events. The operators could request police intervention when needed. The police also recognized the importance of drug checking for health promotion and authorized the activity. However, it must be emphasized that the operators worked independently of the police, because the aim of harm reduction is not punitive.

Approximately 15% (based on the admissions registered by the event organizers and participation in the drug-checking service) of the participants voluntarily and anonymously availed the drug-checking service, and consequently 120 samples were tested on-site. This low participation could be explained, in part, by the possible hesitance of the attendees in engaging in drug-checking services due to stigma and various fears, even if not motivated, such as confiscation of substances, removal from the event, and arrest (3). Other aspects could be related to limited effective information regarding the service, event organizers' resistance to advertising drug checking at event locations as they may not wish their events to be associated with drug dealing and consumption, and finally, inadequate visibility of the location of drug checking. Furthermore, the small percentage of attendees who availed the drug-checking service could also be attributed to the great level of confidence the attendees had about the substances they purchased, and 85% of those who availed the facility stated that they believed the contents to be reliable.

### Colorimetric Test Performance and Substances Identified

For the 120 drug samples tested using colorimetric tests, conclusive and inconclusive results were obtained for 75.83 and 24.17% of the samples, respectively. The positive findings mostly included MDMA (33) and ketamine (25) samples. After a period of low availability linked to a lack of precursor chemicals required for its manufacture, MDMA has experienced a revival in recent years (36). It is a synthetic drug that possesses both stimulant and hallucinogenic properties; it is available in a tablet form (often called ecstasy), and powder and crystalline forms of the drug are also available. New MDMA tablet designs, in various colors, shapes, and brand logos, are constantly being introduced into the market. The retail MDMA market is estimated to be worth approximately EUR 0.7 billion (36). The average content of MDMA in tablets has increased in recent years, and high amounts of MDMA in some batches have been linked to negative effects and death (36).

Ketamine was the second most commonly used drug in this study. Over the past two decades, recreational use of ketamine is increasing (36). It is a short-acting dissociative anesthetic obtained in liquid or powder form and may be delivered orally, intranasally, intramuscularly, or intravenously (36). Owing to the dissociative properties of ketamine, its users describe distortion of time and space, visual hallucinations, and “out-of-body” experiences, notably near-death or religious experiences (37).

Cocaine, heroin, and LSD appeared to be used less frequently. The use of NPS was only marginal among our participants, and two samples of mephedrone were found. The popularity of mephedrone increased from 2008, as evidenced by the increased frequency and quantity of mephedrone seizures by police, surpassing the popularity of other NPS (38, 39). It is an amphetamine analog, considered as a prototypical synthetic cathinone structurally and pharmacological related to MDMA (38, 39).

A comparison of the GC/MS results with the conclusive colorimetric test results showed that the exact chemical nature of the substances was identified with good precision, especially that of LSD (100% of samples confirmed). If the on-site results of a substance were classified as “unknown” (29/120), the situation becomes more complex. These samples were classified on-site as inconclusive by drug-checking operators because the reaction of the substance and the reagent presented a color that is not among the color range in the reagent chart. In these cases, the operators strongly advised the participants against using these substances. Interestingly, a majority (72.41%) of the participants who received an “unknown” result in the on-site test reported that they do not intend to take the substance with a lack of information. Assuming that behavioral intention is an immediate determinant of the actual behavior as suggested by the theories of reasoned action and planned behavior, these results are an indication of the effects drug-checking services can have on the behaviors of used (40, 41). The fact that the participants stated they will not use an unexpected substance is particularly relevant, it is extremely difficult for harm-reduction workers to provide objective and evidence-based information to the users without a drug-checking service to determine drug sample content (40).

Regarding the 29 samples classified as unknown in the on-site test, GC/MS detected 7 samples of ketamine, 2 samples of MDMA, 3 samples of amphetamines, 2 samples of mephedrone, 6 samples of mixtures, 5 samples with adulterants as the main compound, 1 sample of methamphetamine, and 1 of debris. Colorimetric reagents showed a good overall performance for samples that present a clear and easily interpretable chromatic variation, when adulterants were absent (LSD) or present in minimal quantity. Colorimetric reagents regularly fail to identify mixtures and the adulterants present in them. It is also important to underline the failure to identify two samples of NPS (mephedrone), which should have been identified with Lieberman reagent.

### Limitations of Using Colorimetric Reagents and Limitations of the Study

The limitations associated with identifying drugs using colorimetric reagents are well documented (21, 30, 42). The factors contributing to inaccurate results include false-positive color reactions and variations in the response caused by differing drug concentrations or salt form. The colorimetric response produced is subjective and its interpretation can vary with the experience of the analyst, and the type or lack of lighting available when the test is performed (30). The effectiveness of these reagents is further reduced by the increasing frequency of mixtures of substances within pills. The predominant color change masks or interferes with the color change induced by other substances, resulting in the failure to identify other substances (30). In the market, where it is common to encounter combinations of substances within pills, and colorimetric tests could fail to identify all illicit substances present. Here, the GC/MS analysis detected adulterants, of which 53 (84.13%) were commonly found as adulterants, whereas 10 (15.87%) were unusual substances, confirming that the drugs used in the recreational setting are at a high risk of contamination with a wide variety of other harmful substances that have the potential to cause adverse health outcomes (3). This is especially dangerous if users consider their pill “safe,” because although a substance is accurately identified, at high concentrations it may be dangerous (30). Although laboratory analysis by GC/MS performed later can help monitor illicit drug supplies at night events, it cannot guide consumers' choice. Therefore, it is desirable that the drug-checking service is equipped with discriminatory methods on-site such as Fourier transform infrared spectroscopy, ultraviolet-visible spectroscopy, or Raman spectroscopy. However, this is often difficult, because harm-reduction agencies have financial limitations in terms of acquiring more expensive technologies. If the use of more discriminatory methods of drug testing is not feasible due to a lack of financial resources, the performance of colorimetric tests should be improved, using a simple smartphone application to identify colors with high precision and accompanying software that will enable to match the results in a searchable database (16). This would help overcome the limitations of the human eye and its subjectivity (16). Moreover, drug checking can be improved by increasing the number of spot-test reagents used, especially when uncertain results are obtained (16).

The drug-checking service examined was limited, in that a low percentage of participants availed the service. Therefore, in the future, this service must be implemented, for example, by advertisements on various media platforms. Furthermore, because of the small number of participants, the results obtained cannot be generalized to other settings nor provide a comprehensive overview of the drugs circulating in Central Italy.

### Conclusions

The present study has a double value, one essentially technical and the other social. The drug-checking service is a public health promotion activity, as it reduces harms through rapid identification of drugs and discrepancies between the expected and actual contents. It is important to provide a relevant and effective public health message that could orient drug users toward informed and health-protecting choices (20, 40) and to establish contact with hard-to-reach populations (43, 44). The drug-checking service described herein achieves these goals, causing a significant change in the users' behavior when they are confronted with an unexpected result. However, a comparison of results obtained using colorimetric tests and forensic discriminative method highlights the failure of on-site testing in recognizing mixtures of substances and the adulterants contained in them.

## Data Availability Statement

The raw data supporting the conclusions of this article will be made available by the authors, without undue reservation.

## Ethics Statement

The studies involving human participants were reviewed and approved by Bioethics Review Board of University of Perugia. Written informed consent from the participants' legal guardian/next of kin was not required to participate in this study in accordance with the national legislation and the institutional requirements.

## Author Contributions

MF and AA carried out drug samples collection and on-site analysis. CC supervised the harm reduction project. AG analyzed the data. MB verified the analytical methods. AN carried out sample preparation and GC/MS analysis. CG carried out GC/MS analysis and wrote the manuscript with input from all authors. All authors read and approved the final manuscript.

## Conflict of Interest

The authors declare that the research was conducted in the absence of any commercial or financial relationships that could be construed as a potential conflict of interest.
