# Supplementary material for: Drug Checking as Strategy for Harm Reduction in Recreational Contests: Evaluation of Two Different Drug Analysis Methodologies
Source: Front Psychiatry. 2021 Feb 22;12:596895. doi: 10.3389/fpsyt.2021.596895 (PMC7938318; doi:10.3389/fpsyt.2021.596895)
Supplement: Supplementary file 1 [file Data_Sheet_1.PDF]

ON SITE FORM

DATA _____________ GENERE: _____________ ETÀ _________
(DATE) (*GENDER)* (*AGE*)

BorgoRete - Soc. Coop. Soc.
Sede legale: via F.lli Cairoli, 24 - 06125 Perugia
Tel. 075/5145100 – Fax 075/5004584
P.I. 00589560549
Iscr.Albo Società Cooperative a Mutualità Prevalente n. A146423

ON SITE FORM

SOSTANZA ATTESA ____
(*EXPECTED SUBSTANCE*)

HAI CONSUMATO LA SOSTANZA PRIMA DEL TEST? SÍ ☐ NO ☐
(*DID YOU CONSUME THE SUBSTANCE BEFORE THE TEST*?) *(YES)* *(NO)*

CHE RISULTATO TI ASPETTI DAL TEST COLORIMETRICO?
(*WHAT RESULT DO YOU EXPECT FROM THE COLORIMETRIC TEST?)*

- ☐ LA SOSTANZA CHE HO COMPRATO (*THE SUBSTANCE I BOUGHT*)
- ☐ UN ADULTERANTE/TAGLIO (*AN ADULTERANT*)
- ☐ UN'ALTRA SOSTANZA (*ANOTHER SUBSTANCE*)

DOPO AVER RICEVUTO INFORMAZIONI A PROPOSITO DEL DRUG CHECKING, ACCETTI DI CONSEGNARE UN PICCOLA FRAZIONE DELLA TUA SOSTANZA PER ESSERE ANALIZZATA ON SITE E SUCCESSIVAMENTE IN LABORATORIO? SÍ ☐ NO ☐
(AFTER RECEIVING COMPLETE INFORMATION ABOUT DRUG CHECKING DO YOU AGREE TO DELIVER A SMALL FRACTION OF YOUR SUBSTANCE TO BE ANALYZED ON SITE AND LATER IN THE LABORATORY? YES ☐ NO ☐)

ORA CHE CONOSCI IL RISULTATO ASSUMERAI LA SOSTANZA? SÍ ☐ NO ☐
(*NOW THAT YOU KNOW THE TEST RESULT ARE YOU GOING TO CONSUME THE SUBSTANCE?* YES ☐ NO ☐)

BorgoRete - Soc. Coop. Soc.
Sede legale: via F.lli Cairoli, 24 - 06125 Perugia
Tel. 075/5145100 – Fax 075/5004584
P.I. 00589560549
Iscr.Albo Società Cooperative a Mutualità Prevalente n. A146423

ON SITE FORM

TEST COLORIMETRICO
(COLORIMETRIC TEST)

DESCRIZIONE CAMPIONE______________________
(SAMPLE DESCRIPTION)

**Reagente (Colorimetric Reagents)**	**Positive**	**No reaction**
Marquis		
Mecke		
Mandelin		
Lieberman		
Scott		

RISULTATO DEL TEST COLORIMETRICO
(COLORIMETRIC TEST RESULT)

☐ Conclusivo ___________________ ☐Non conlusivo___________________
Conclusive *(inconclusive)*

*Sostanza identificata*______________________________________
(identified substance)

BorgoRete - Soc. Coop. Soc.
Sede legale: via F.lli Cairoli, 24 - 06125 Perugia
Tel. 075/5145100 – Fax 075/5004584
P.I. 00589560549
Iscr.Albo Società Cooperative a Mutualità Prevalente n. A146423
