# Supplementary material for: Drug Checking as Strategy for Harm Reduction in Recreational Contests: Evaluation of Two Different Drug Analysis Methodologies
Source: Front Psychiatry. 2021 Feb 22;12:596895. doi: 10.3389/fpsyt.2021.596895 (PMC7938318; doi:10.3389/fpsyt.2021.596895)
Supplement: Supplementary file 2 [file Data_Sheet_2.PDF]

FOR FORENSIC LABOATORY DELIVERY

DATA ____________
(DATE)

RISULTATO DEL TEST COLORIMETRICO
(COLORIMETRIC TEST RESULT)

☐ Conclusivo/sostanza identificata___________________ ☐Non conlusivo___________________
(Conclusive/identified substance) (inconclusive)

BorgoRete - Soc. Coop. Soc.
Sede legale: via F.lli Cairoli, 24 - 06125 Perugia
Tel. 075/5145100 – Fax 075/5004584
P.I. 00589560549
Iscr.Albo Società Cooperative a Mutualità Prevalente n. A146423
